# Neutrophils and NADPH Oxidases Are Major Contributors to Mild but Not Severe Ischemic Acute Kidney Injury in Mice

**DOI:** 10.3390/ijms25052948

**Published:** 2024-03-03

**Authors:** Csaba Révész, Tamás Kaucsár, Mária Godó, Krisztián Bocskai, Tibor Krenács, Attila Mócsai, Gábor Szénási, Péter Hamar

**Affiliations:** 1 Institute of Translational Medicine, Semmelweis University, 1094 Budapest, Hungary; kecsan-revesz.csaba@med.semmelweis-univ.hu (C.R.); kaucsar.tamas@med.semmelweis-univ.hu (T.K.); bocskaister@gmail.com (K.B.); hamar.peter@semmelweis.hu (P.H.); 2 Department of Pathology and Experimental Cancer Research, Semmelweis University, 1085 Budapest, Hungary; krenacs.tibor@med.semmelweis-univ.hu; 3 Department of Physiology, Semmelweis University, 1094 Budapest, Hungary; mocsai.attila@semmelweis.hu

**Keywords:** acute kidney injury, ischemia–reperfusion injury, reactive oxygen species, oxidative stress, NADPH oxidase, xanthine oxidoreductase, RNA interference, Mcl-1^ΔMyelo^, neutrophil deficiency

## Abstract

Upregulation of free radical-generating NADPH oxidases (NOX), xanthine oxidoreductase (XOR), and neutrophil infiltration-induced, NOX2-mediated respiratory burst contribute to renal ischemia–reperfusion injury (IRI), but their roles may depend on the severity of IRI. We investigated the role of NOX, XOR, and neutrophils in developing IRI of various severities. C57BL/6 and Mcl-1^ΔMyelo^ neutrophil-deficient mice were used. Oxidases were silenced by RNA interference (RNAi) or pharmacologically inhibited. Kidney function, morphology, immunohistochemistry and mRNA expression were assessed. After reperfusion, the expression of NOX enzymes and XOR increased until 6 h and from 15 h, respectively, while neutrophil infiltration was prominent from 3 h. NOX4 and XOR silencing or pharmacological XOR inhibition did not protect the kidney from IRI. Attenuation of NOX enzyme-induced oxidative stress by apocynin and neutrophil deficiency improved kidney function and ameliorated morphological damage after mild but not moderate/severe IRI. The IR-induced postischemic renal functional impairment (BUN, Lcn-2), tubular necrosis score, inflammation (TNF-α, F4/80), and decreases in the antioxidant enzyme (GPx3) mRNA expression were attenuated by both apocynin and neutrophil deficiency. Inhibition of NOX enzyme-induced oxidative stress or the lack of infiltration by NOX2-expressing neutrophils can attenuate reperfusion injury after mild but not moderate/severe renal IR.

## 1. Introduction

Acute kidney injury (AKI) is a rapid loss of renal function that frequently leads to death in intensive care units [1]. The leading cause of AKI is ischemia–reperfusion injury (IRI). IRI involves complex pathophysiological mechanisms, including disturbances in energy metabolism and redox imbalance [2]. The morphological manifestation of IRI is acute tubular necrosis (ATN), characterized by tubular epithelial cell damage: loss of brush border, dilation of tubules, and cell death [3]. IRI leads to endothelial dysfunction and inflammation [2,4]. Blood urea nitrogen (BUN) and lipocalin-2 (Lcn-2) concentrations are specific early markers of kidney injury [5]. Inflammation of the postischemic tissue through the infiltration of neutrophils/macrophages can be monitored by neutrophil-specific myeloperoxidase (MPO), while F4/80 (also named EGF-like module-containing mucin-like hormone receptor-like 1, EMR1) is a marker of macrophage populations. Tumor necrosis factor alpha (TNF-α) is a conventional marker of tissue inflammation [6]. Important indicators of oxidative stress are the extracellular antioxidant enzyme glutathione peroxidase 3 (GPx3), the cellular antioxidant enzyme heme oxygenase 1 (HO-1), and its transcriptional regulator nuclear factor erythroid 2-related factor 2 (NRF2) [7].

Tight regulation of generating and eliminating reactive oxygen species (ROS) contributes to maintaining redox homeostasis [8]. Superoxide is produced mainly by the membrane-bound nicotinamide adenine dinucleotide phosphate oxidase (NADPH oxidase, NOX) family enzymes [9,10]. NOX enzymes are upregulated after IR and are major contributors to postischemic ROS generation [4,11]. The beneficial effects of inhibiting the NOX enzyme-induced oxidative stress by apocynin confirmed the role of NOX enzymes in the pathogenesis of IRI only in rats, especially in the long term after reperfusion [12,13]. However, the contribution of the most abundant, kidney-specific NOX4 isoform to renal IR injury is still controversial [14,15].

Xanthine oxidoreductase (XOR) has xanthine oxidase (XO) or dehydrogenase (XDH) activity and produces ROS during reperfusion [16]. The beneficial effects of XOR-inhibitor allopurinol and its active metabolite oxypurinol [17] were demonstrated in renal IRI [18]. In contrast, XOR also generates nitric oxide as a nitrate/nitrite reductase, contributing to vasodilatation, which is considered beneficial under ischemic/hypoxic conditions [19].

Although several antioxidants were beneficial during preservation for improving graft function [20,21], no drugs successfully prevent or treat AKI. Often, conflicting results are reported regarding the possible therapeutic potential of inhibitors against oxidative stress in general, or against specific ROS sources. One possible reason for the contradictory observations may be that ROS formation is a relatively significant source of injury after mild AKI but may be lower after severe ischemia, as the enzymes involved are also severely damaged. Furthermore, when the kidney tissue is seriously injured by prolonged ischemia, oxidative stress may cause minor additional damage, as several other deleterious mechanisms are also activated.

Therefore, the main hypothesis of the study was that the protection against oxidative stress explicitly induced by free radical-generating enzymes, such as NADPH oxidase (NOX) enzymes and xanthine oxidoreductase (XOR), strongly depends on the severity of ischemic damage. The hypothesis was tested at three durations of ischemia using three approaches: first, treatment with apocynin, which can attenuate oxidative stress induced by NOX enzymes, or oxypurinol, which is an XOR inhibitor; second, testing the effects of renal ischemic injury in neutrophil-deficient mice, since infiltrating neutrophils are an essential source of NOX2-derived reactive oxygen species; and third, by silencing XOR or NOX4, the most prevalent renal NOX enzyme gene. 

## 2. Results

### 2.1. Severe Ischemia–Reperfusion Impaired Kidney Function and Morphology

**Figure 1:**
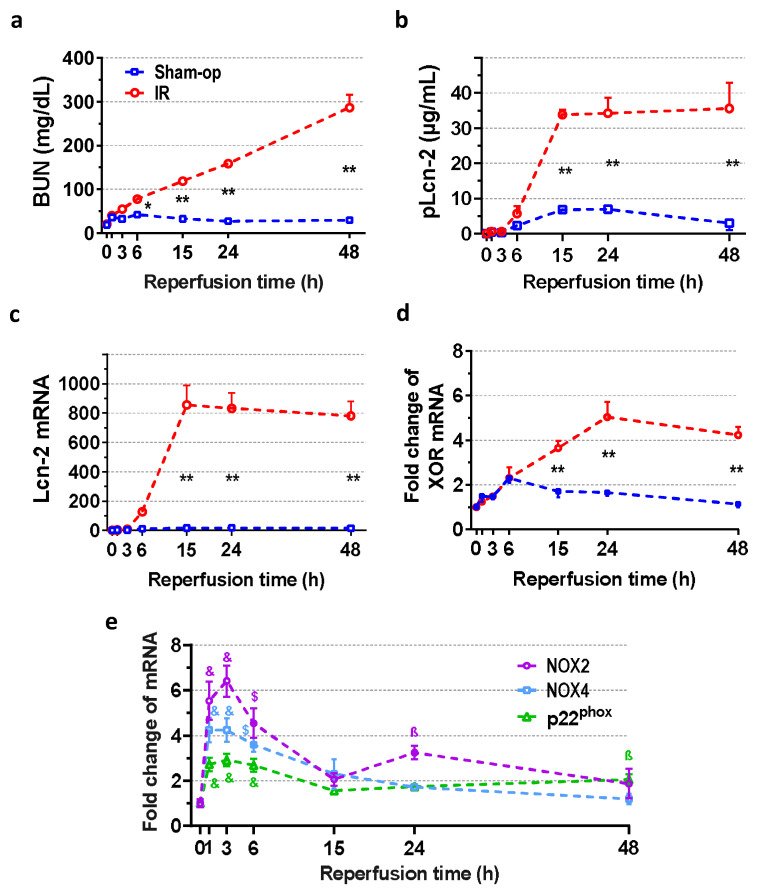
Time course of postischemic kidney function and expression of oxidase enzymes after 30 min of ischemia. Functional marker BUN indicates an early onset of oxidative damage as NOX is upregulated. The elevation of the injury marker Lcn-2 between 6 and 15 h implies ongoing parenchymal injury. (**a**) Blood urea nitrogen (BUN), (**b**) lipocalin-2 (Lcn-2) plasma concentration, (**c**) renal Lcn-2 gene expression, (**d**) XOR mRNA expression and (**e**) NOX mRNA expression. Sham-op: sham-operated group. IR: ischemia–reperfusion group. (**a**–**d**) * *p* < 0.05, ** *p* < 0.0001 vs. sham-op. (**e**) ^ß^ *p* < 0.05, ^$^ *p* < 0.001, ^&^ *p* < 0.0001 vs. 0 h group. Two-way ANOVA with Sidak post hoc test. *n* = 5/group/time.

**Figure 2:**
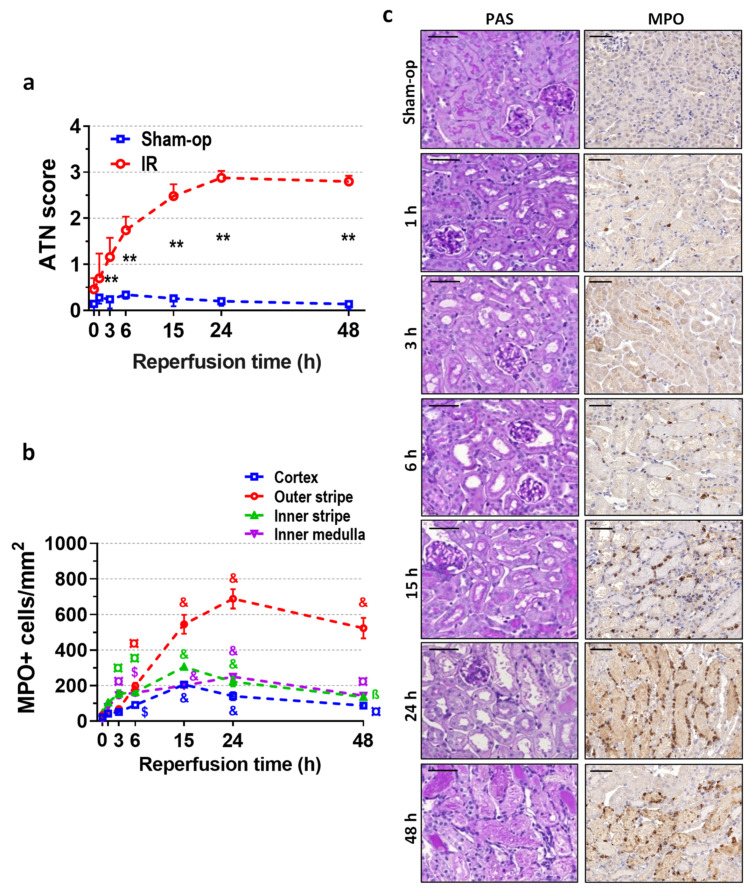
Postischemic time course of renal morphology and neutrophil infiltration after 30 min of ischemia. Similar to function, morphology indicates an early start of oxidative injury resulting in neutrophil infiltration. (**a**) Acute tubular necrosis (ATN) score, (**b**) MPO+ cell count/mm^2^, (**c**) periodic acid-Schiff (PAS) staining, and myeloperoxidase (MPO) immunohistochemistry. Scale bar: 50 μm. Sham-op: sham-operated group. IR: ischemia–reperfusion group. (**a**) ** *p* < 0.0001 vs. sham-op. (**b**) ^ß^ *p* < 0.05, ^¤^ *p* < 0.01, ^$^ *p* < 0.001, ^&^ *p* < 0.0001 vs. 0 h group. Two-way ANOVA with Sidak post hoc test. *n* = 5/group/time.

In the sham-operated animals, BUN and Lcn-2 concentrations, renal Lcn-2 mRNA expression, and acute tubular necrosis (ATN) score were similar to those of control groups previously published by our laboratory (Figure 1a–c and Figure 2a) after 30 min of ischemia [5]. Compared to the control value (19.2 ± 2.0 mg/dL), BUN concentration gradually increased to 77.9 ± 4.5 mg/dL at 6 h and to 286.7 ± 29.8 mg/dL at 48 h (Figure 1a) after 30 min of ischemia. Renal Lcn-2 mRNA was upregulated from 6 h and reached a 100-fold plateau at 15 h (Figure 1c), while the plasma concentration of Lcn-2 increased 10-fold compared to the sham-operated group (Figure 1b).

Morphological signs of ATN were present from 3 h onwards, and the ATN score increased to 3 at 24 and 48 h post-reperfusion (Figure 2a,c). Immunohistochemistry for MPO revealed ongoing neutrophil infiltration in the inner medulla and the inner stripe of the outer medulla as early as 3 h after 30 min of ischemia (Figure 2b,c). Infiltrating neutrophils appeared in the outer stripe and the cortex after 6 h, and their interstitial presence around the injured tubules was pronounced at 15 h post-ischemia (Figure 2c). After the 24 h peak, a slight decrease was revealed in the infiltration. At 24 h post-ischemia, dilated tubules with flattened epithelial cells dominated the morphology of the postischemic kidney (Figure 2c). The strongest infiltration was detected in the outer stripe of the outer medulla, where the number of MPO-positive cells was the highest at 24 h, and neutrophils were still present around the necrotic tubules at 48 h.

### 2.2. Rapid Renal NOX and Delayed XOR Upregulation after Reperfusion

Oxidase enzymes (XOR and NOX) mRNA was low in the sham-operated kidneys. A 2.3-fold increase in XOR mRNA was found at 6 h after sham operation. Compared with sham-operated kidneys, XOR mRNA began to increase at 15 h post-ischemia, reached a maximum 5-fold increase at 24 h, and was still elevated at 48 h after severe (30-min) ischemia (Figure 1d). On the other hand, expression of NADPH oxidases increased maximally as early as 1 to 3 h and slowly recovered by 15 h post-ischemia, but remained elevated at 24 or 48 h (Figure 1e). Furthermore, a small second peak was observed in the NOX2 gene expression. NOX2, NOX4, and p22^phox^ mRNA expression increased maximally 6-, 4-, and 3-fold at 3 h after reperfusion.

### 2.3. Pharmacological Inhibition of NOX Enzymes but Not XOR Protected against Mild but Not Moderate or Severe Renal Ischemia

**Figure 3:**
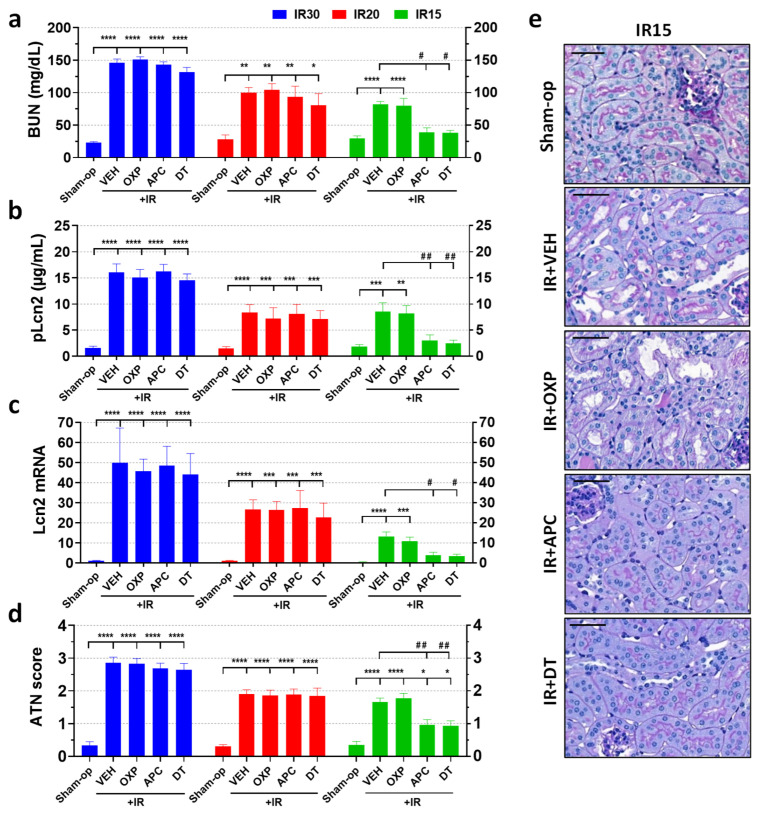
The non-selective NADPH oxidase inhibitor, apocynin, attenuated kidney injury and morphological damage after IR, but its protective effect depended on the severity of ischemia. Inhibition of xanthine oxidoreductase (XOR) activity or expression was ineffective, and kidney-specific NOX4 did not contribute to renal IRI. (**a**) BUN, (**b**) Lcn-2 plasma concentration, (**c**) renal Lcn-2 gene expression, (**d**) ATN score, (**e**) renal histology (PAS staining; scale bar: 50 μm). Sham-op: sham-operated group. IR: ischemia–reperfusion group. IR15/20/30 refers to the length (min) of ischemia. VEH: vehicle, APC: apocynin, OXP: oxypurinol, DT: double treatment. * *p* < 0.05, ** *p* < 0.01, *** *p* < 0.001, **** *p* < 0.0001 vs. sham-op. # *p* < 0.05, ## *p* < 0.01 vs. IR + VEH. One-way ANOVA with Dunnett post hoc test (IR30: *n* = 6-5-7-7-6; IR20: *n* = 5-7-7-6-6; IR15: *n* = 6-8-7-7-7).

Compared to the sham-operated animals, renal ischemia of various durations increased all renal injury markers (BUN and Lcn-2 concentrations, Lcn-2 renal gene expression and ATN score) in proportion to the severity of ischemia (Figure 3a–e). Apocynin, a non-selective NOX enzyme inhibitor, improved renal function and morphology at 24 h after mild (15 min) but not moderate (20 min) or severe (30 min) ischemia. Oxypurinol, an XOR inhibitor, did not improve renal function and morphology after renal ischemia of either severity. Furthermore, a combination of the two inhibitors, apocynin and oxypurinol, was similarly effective as apocynin alone.

### 2.4. XOR and NOX4 Silencing Did Not Protect Renal Function Post-Ischemia

**Figure 4:**
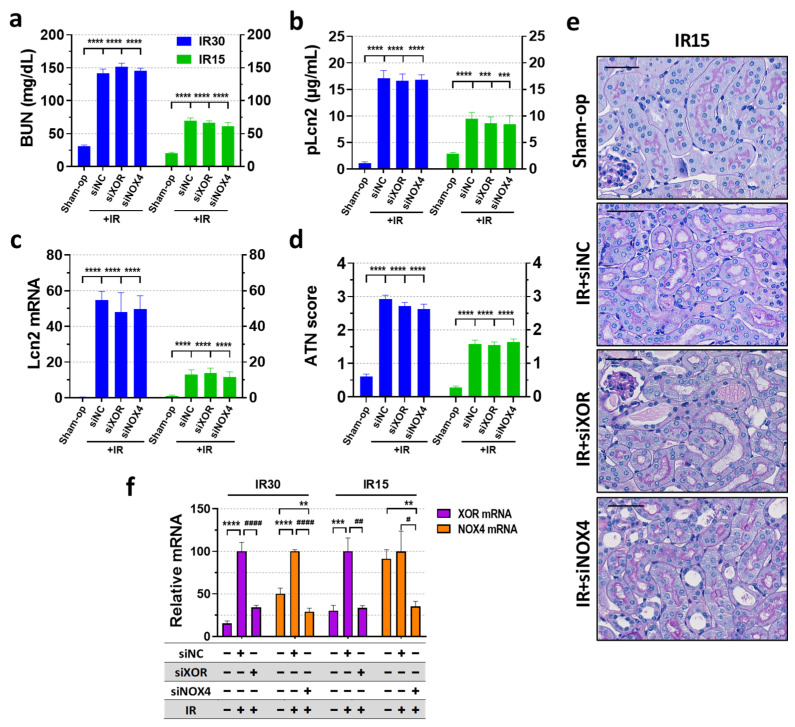
SiRNA-mediated knockdown of XOR and NOX4 genes failed to alleviate functional and morphological kidney injury, regardless of the severity of ischemia. (**a**) BUN, (**b**) Lcn-2 plasma concentration, (**c**) renal Lcn-2 gene expression, (**d**) ATN score, (**e**) renal histology (periodic acid-Schiff (PAS) staining; scale bar: 50 μm), (**f**) RNAi knockdown efficacy. Sham-op: sham-operated group. IR: ischemia–reperfusion group. IR15/30 refers to the length (min) of ischemia. ** *p* < 0.01 *** *p* < 0.001, **** *p* < 0.0001 vs. Sham-op. # *p* < 0.05, ## *p* < 0.01, #### *p* < 0.0001 vs. IR + VEH. One-way ANOVA with Dunnett post hoc test (IR30 *n* = 7-8-9-9; IR15 *n* = 8-8-8-7).

XOR mRNA expression increased more than 5-fold and 3-fold at 24 h after severe (30 min) or mild (15 min) renal ischemia, respectively (Figure 4f). Transfection with XOR siRNA completely (15 min) or partially (30 min) reversed the ischemia-induced increase in XOR mRNA expression with a knockdown efficiency of 66% in both cases. Renal NOX4 expression approx. doubled after 30 min of ischemia but was not influenced after 15 min of ischemia (Figure 4f). NOX4 siRNA silencing inhibited the ischemia-induced increase in NOX4 mRNA. SiRNA inhibition reduced NOX4 mRNA below the control level with over 70% knockdown efficiency in the 30 min IR experiment, while silencing efficiency was 65% in the 15 min IR experiment.

In the RNAi experiments, renal IR significantly increased all renal injury markers (BUN, Lcn-2 concentrations and Lcn-2 mRNA) and morphological damage (ATN score) at 24 h after severe (30 min) and mild (15 min) ischemia (Figure 4a–e), similarly to that of the enzyme inhibition experiment. Despite successful silencing of XOR and NOX4, kidney function was not improved after either severe (30 min) or mild (15 min) renal ischemia (Figure 4a–c). Histological sections of the siRNA-silenced groups showed similar morphological damage in the kidneys to that of the control IR animals (Figure 4d,e).

### 2.5. Pharmacological Inhibition of NOX by Apocynin Increased Antioxidant Defense and Mitigated Inflammation after Mild Renal Ischemia

**Figure 5:**
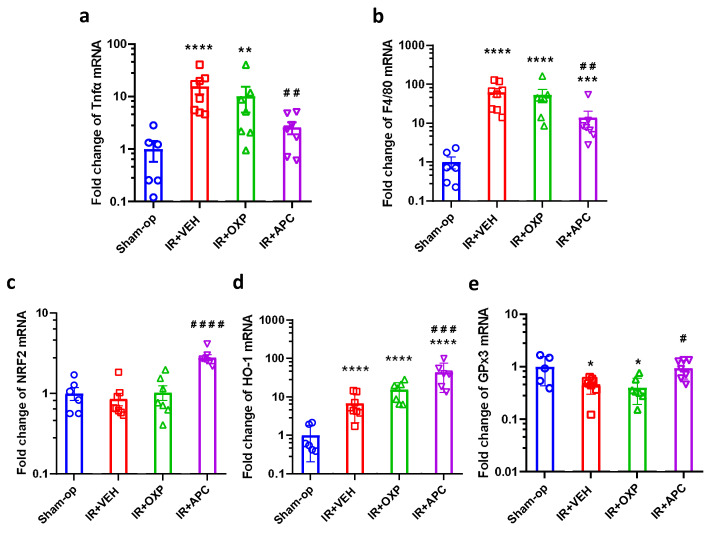
The decrease in TNF-α and F4/80 expression indicated less inflammation after NADPH oxidase inhibition with apocynin after mild ischemia–reperfusion. Oxidative stress-related gene upregulation by apocynin demonstrated greater protection against oxidative stress. (**a**) TNF-α, (**b**) F4/80, (**c**) NRF2, (**d**) HO-1, (**e**) GPx3 mRNA expression. Sham-op: sham-operated group. IR: ischemia–reperfusion group. VEH: vehicle, APC: apocynin, OXP: oxypurinol, * *p* < 0.05, ** *p* < 0.01, *** *p* < 0.001, **** *p* < 0.0001 vs. Sham-op. # *p* < 0.05, ## *p* < 0.01, ### *p* < 0.001, #### *p* < 0.0001 vs. IR + VEH. One-way ANOVA with Dunnett post hoc test (sham *n* = 6, IR + VEH *n* = 8, IR + inhibitor groups *n* = 7).

When kidneys were subjected to mild IRI, apocynin attenuated functional impairment and morphological damage after mild (15 min) ischemia (Figure 4a–e). Furthermore, double treatment was as effective as apocynin alone, i.e., inhibition of XOR had no additive effect. This protective effect of apocynin was observed in the inflammatory and antioxidant gene expression. Mild renal IR increased mRNA expression of TNF-α by 15-fold, F4/80 by 60-fold, and HO-1 by 7-fold but decreased GPx3 to 50% of the control and did not alter NRF2 mRNA expression compared to the sham-operated group (Figure 5a–e). Apocynin administration increased renal NRF2 expression by 2.8-fold and IR-induced HO-1 expression from 7-fold to 44-fold in the postischemic kidneys of mice subjected to mild IR compared with that in the vehicle-treated group (Figure 5c,d). Apocynin decreased the mRNA expression of TNF-α and F4/80 (Figure 5a,b) and prevented the decrease in GPx3 gene expression in the kidney (Figure 5e). 

### 2.6. Neutrophil-Deficient Bone Marrow Chimeric Mice Are Protected from Mild Renal IRI 

**Figure 6:**
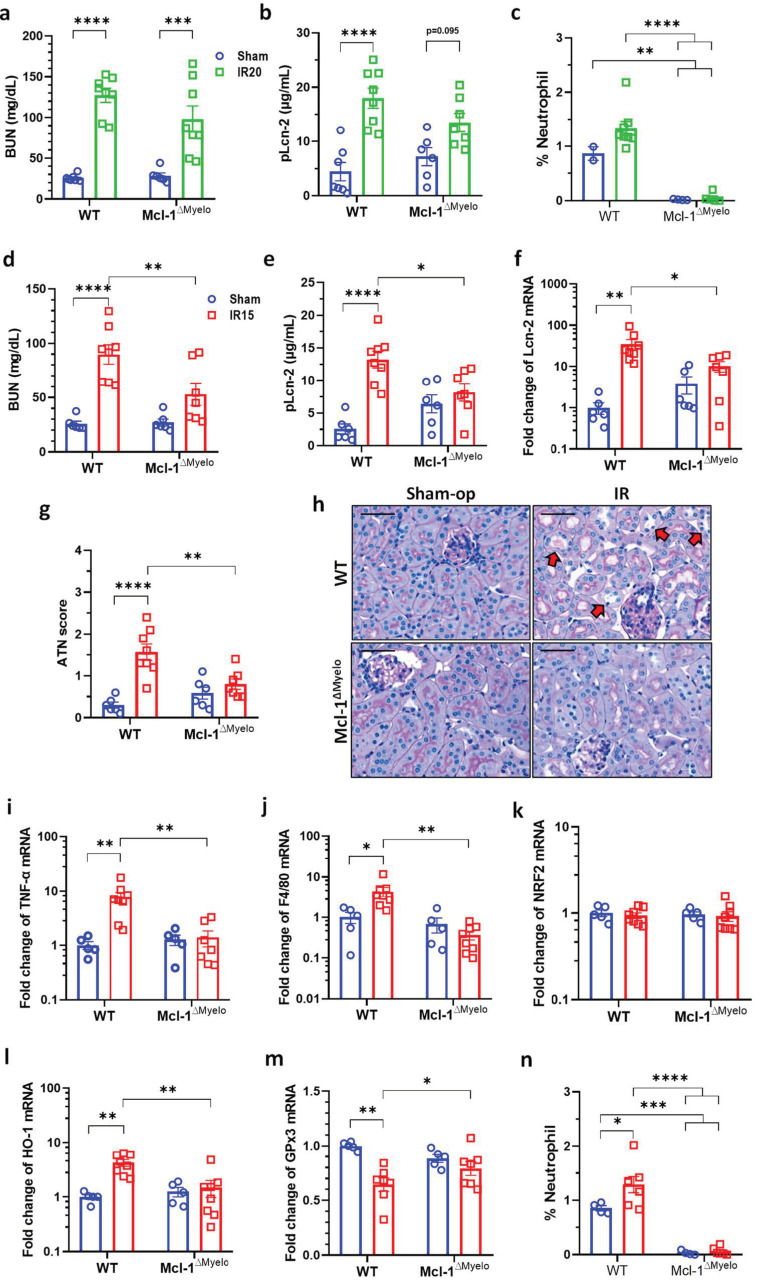
Neutrophil-deficient bone marrow chimeric mice are protected from mild renal ischemia–reperfusion injury (IRI). (**a**) BUN, (**b**) Lcn-2 plasma concentration and (**c**) the proportion of neutrophils in the blood after moderate (20 min) renal I/R. (**d**) BUN, (**e**) plasma Lcn-2 concentration, (**f**) renal Lcn-2 expression, (**g**) ATN score, (**h**) renal histology (PAS staining; scale bar: 50 μm), (**i**) TNF-α, (**j**) F4/80, (**k**) NRF2, (**l**) HO-1, (**m**) GPx3 mRNA expression, (**n**) the proportion of neutrophils in the blood after moderate (20 min) renal I/RN. Neutrophil deficiency was verified by flow cytometry. Sham-op: sham-operated group. IR: ischemia–reperfusion group. WT: wild-type, Mcl-1^ΔMyelo^: neutrophil-deficient bone marrow chimeric mice. * *p* < 0.05, ** *p* < 0.01, *** *p* < 0.001, **** *p* < 0.0001. Two-way ANOVA with Sidak post hoc test ((**a**–**d**) renal function/histology: sham groups *n* = 6; WT IR *n* = 8; Mcl-1^ΔMyelo^ IR *n* = 7; (**f**–**j**) mRNA expression: sham *n* = 5; IR *n* = 8; (**k**) FACS: sham groups *n* = 4; WT IR *n* = 7; Mcl-1^ΔMyelo^ IR *n* = 6).

Neutrophil granulocytes were practically absent from the circulation of conditional Mcl-1-deficient bone marrow-transplanted (Mcl-1^ΔMyelo^) chimeric mice. The proportion of neutrophils increased in the blood of wild-type (WT) mice at 24 h after IR (Figure 6c,n).

After 20 min ischemia, BUN and Lcn-2 concentrations were increased in WT compared to sham-operated animals (Figure 6a,b). Similar increases were observed in the neutrophil-deficient Mcl-1^ΔMyelo^ mice after 20 min ischemia (Figure 6a,b). In contrast, changes in BUN and Lcn-2 concentrations, renal Lcn-2 mRNA expression, and ATN score were significantly lower in neutrophil-deficient than in WT mice after mild, 15 min renal ischemia (Figure 6d–g). Histological sections showed preserved morphology in the kidneys of the neutrophil-deficient mice when compared to WT animals (Figure 6h, arrows). TNF-α, F4/80 and HO-1 expression increased, and GPx3 decreased, whereas NRF2 remained unaffected in WT mice after mild ischemia (Figure 6i–m). In contrast, none of these mRNAs were altered in the kidneys of neutrophil-deficient mice (Figure 6i–m). Histological damage was also reduced in the kidneys of Mcl-1^ΔMyelo^ mice (Figure 6h).

## 3. Discussion

We have shown that apocynin and neutrophil deficiency protected against only mild, but not moderate or severe, renal ischemic injury, while silencing XOR or the kidney-specific NOX4 gene were not protective even against mild renal ischemic injury. The novelty of the study is that, firstly, the reduction of oxidative stress mediated by NOX has a protective effect only after mild renal ischemia/reperfusion; secondly, neutrophil deficiency was also protective, the effect of which was also manifested exclusively after mild renal ischemia/reperfusion.

There is a strong consensus on the role of oxidative stress in IRI [21,22]. However, numerous enzymes can form ROS from molecular oxygen after reperfusion. The contribution of the individual sources of ROS to the pathophysiology of renal IRI remains to be fully elucidated. We monitored the expression of the main enzymes of oxidative stress, such as NADPH oxidases (NOX2, NOX4, and a common NADPH subunit, p22^phox^) and xanthine oxidoreductase (XOR) in the kidney. Our time–course data revealed a rapid but transient increase in the expression of NOX isozymes and strong and long-lasting upregulation of XOR, suggesting a possible role of these enzymes in renal oxidative stress-related injury. Ischemia of various durations (15, 20, and 30 min) resulted in renal injury of proportional severity. BUN and ATN score time course indicated a continuously progressing injury after reperfusion. MPO immunohistochemistry showed that neutrophil infiltration began 3 h after reperfusion but was substantial by 24 h, as demonstrated by mRNA expression of the macrophage marker F4/80.

Many studies proved that suppression of ROS production is beneficial by decreasing oxidative stress in different organs [21,22]. XOR, a harmful ROS producer and a source of extracellular superoxide, is considered to contribute to the inflammatory response through TLR4-mediated neutrophil activation in IRI [23]. Allopurinol, an XOR inhibitor, reduced oxidative stress in hypoxia/ischemia in the kidney [24]. However, in our study, neither oxypurinol, the major and active metabolite of allopurinol, nor silencing XOR expression alleviated renal injury after any duration of ischemia. These results suggest that XOR is not a major source of deleterious oxidative stress and consequent reperfusion injury in mice, similar to observations in rats [25,26]. A single knockdown of NOX4 by RNAi confirmed that NOX4 is not a significant contributor to kidney injury, as shown before [27]. Nonetheless, a recent study showed that oxypurinol, at the same dose and in the same mouse strain as in the present study, prevented kidney injury induced by 30 min right-side renal IR and left nephrectomy [18]. However, the authors used 2.5 mL DMSO for dissolving oxypurinol, and DMSO possessed protective effects even at the dose of 0.1 mL in spinal cord IRI in rabbits [28]. Therefore, it cannot be excluded that the weak protective effects of DMSO and oxypurinol were additive, indicating a false-protective effect for oxypurinol.

Apocynin pretreatment improved kidney function and morphology, but only after mild (15 min) renal ischemia. The results were similar in the groups double-treated with apocynin and oxypurinol, supporting the idea that XOR inhibition was not protective.

IR induces TNF-α production, mainly in the injured tubular epithelial and endothelial cells. Releasing TNF-α promotes neutrophil infiltration, having a major role in the activation of the postischemic response of the innate immune system [29]. Nevertheless, TNF-α is an adequate marker for monitoring IR injury. The other central cell type of innate immunity in the pathogenesis of IR is macrophages. In the initial period of reperfusion, pro-inflammatory macrophages infiltrate the injured parenchyma and aggravate the postischemic tissue damage [30]. Macrophage-specific inflammatory marker F4/80 is a competent marker to examine inflammatory status in IR [31]. Renal improvement by apocynin in response to postischemic oxidative damage was seen in the reduction in the expression of inflammation-related markers, such as the pro-inflammatory cytokine TNF-α and macrophage marker F4/80.

NRF2 is involved in antioxidant defense by promoting the transcription of antioxidant response element (ARE) genes, e.g., heme oxygenase (HMOX)-1, NAD(P)H quinone oxidoreductase (NQO)-1, and thioredoxin reductase (TXNRD)-1 [32,33]. Hypoxia is one of the main factors that induce HO-1 expression, the product of the HMOX-1 gene. HO-1, the inducible heme oxygenase isoform, is a protective mediator in many renal pathologies [34]. Apocynin can increase NRF2 protein expression despite decreasing oxidative stress [35,36], as also found in the present study. This effect is associated with p62-dependent regulation of NRF2 expression independent of oxidative stress [37]. As NRF2 regulates HO-1, apocynin initiates the nephroprotective effect of HO-1 by increasing NRF2 expression, which explains the good protective effect of apocynin after IR [32,38]. The increase in HO-1 gene expression without a change in NRF2 mRNA expression can be explained by the different kinetics of the two genes. NRF2 expression appears to have normalized 24 h after mild ischemia, but postischemic HO-1 expression is still elevated simultaneously. In addition, inhibition of NRF2 degradation can also contribute to the maintenance of increased HO-1 expression for extended periods. Glutathione peroxidase family members are essential factors of antioxidant defense. GPx3, the plasma glutathione peroxidase, was found to be a potent biomarker of oxidative stress in renal IRI due to its robust relation to oxidative stress-related, immune response-related, and apoptosis-related signaling pathways [39]. The concentration of the extracellular ROS-scavenging GPx3 isoform was reduced in oxidative stress [40], as also found in this study. Treatment with apocynin restored GPx3 mRNA expression to the control level. The induction of GPx3 can be one of the antioxidant mechanisms of apocynin since GPx3 depletion induces NOX2 and can promote kidney fibrosis through a NOX2/ROS/p38 MAPK pathway [41]. 

Inhibition of NOX-related oxidative stress by apocynin ameliorated kidney function and preserved morphology against mild, 15 min ischemia in our IRI model. Nevertheless, when renal ischemia lasted for 20 or 30 min, no protection was observed after the inhibition of NADPH oxidase-related oxidative stress, suggesting that ischemic cell function was so severely impaired that inhibition of oxidative stress was insufficient for any improvement. These results of NOX inhibition indicate a translational relevance for preventing the kidney from IRI due to minor ischemia but also point to the limitations of inhibiting ROS production in renal IR. After a prolonged ischemic event, severe IR seems to result in predominantly NOX-independent parenchymal injury.

The infiltrating neutrophils have a dual role in the pathology of IRI: they contribute to the inflammatory response in IR as a part of innate immunity and are responsible for oxidative damage as ROS generators via NOX2-mediated oxidative burst. Neutrophil infiltration considerably increased after IR, shown by continuously increasing MPO immunohistochemical staining after reperfusion. Infiltration began 3 h after reperfusion and persisted for 48 h in the postischemic kidney [42]. In our study, the NOX enzymes increased rapidly at mRNA levels within 1 h after ischemia. This response was also demonstrated at protein levels in a swine IR model and was considered complement-dependent [43]. We found that the mRNA expression of renal NOX2, NOX4, and p22^phox^ was the greatest at 3 h, and a second elevation of NOX2 expression was observed at 24 h of reperfusion when neutrophil infiltration was the highest in the injured kidney.

These results confirm a consecutive, two-wave oxidative stress in renal IR. The early oxidative stress is driven by a rapid postischemic increase in local NOX expression, resulting in initial oxidative damage, followed by the recruitment of neutrophils, which infiltrate the renal interstitium and contribute to a second wave of postischemic kidney injury by the NOX2-mediated respiratory burst, which was found by others also in hepatic IRI [44]. The most significant and extended neutrophil infiltration was revealed in the outer stripe of the medulla, which points to the localization of the most severe parenchymal injury after IR.

In the kidney, the basal expression of the NOX2 gene is lower than that of other oxidases (two orders of magnitude lower than NOX4 or XOR; our unpublished data). However, there are two sources of NOX2: the local parenchymal and the phagocytic NOX2, mainly in infiltrating neutrophils. The postischemic activity of these two NOX2 populations shifted during the reperfusion period, as demonstrated by the time course of renal NOX2 mRNA expression and MPO immunohistochemistry. RNAi-based silencing of the two populations of NOX2 was not performed in this study as silencing is ineffective in peripheral leukocytes in vivo, e.g., in neutrophils [45]. The negative results after NOX4 RNAi and positive results with apocynin point to the importance of ROS generation by infiltrating neutrophils in the pathomechanism of renal IRI. Their high capacity for superoxide generation makes neutrophils essential players in the pathophysiology of various diseases [46]. Indeed, neutrophil-deficient chimeric mice showed strong protection against mild IRI, confirming the involvement of neutrophils in the IR-mediated AKI. In contrast to WT animals, renal HO-1, F4/80 and TNF-α mRNA expression did not increase after IR in neutrophil-deficient mice. As a result, GPx3 expression remained unaffected, contrary to WT mice. Apocynin produced similar effects to neutrophil deficiency. Apocynin can have two mechanisms of action. One is that apocynin inhibits the local renal NADPH oxidases early in reperfusion which reduces neutrophil infiltration and NOX2 in neutrophils late in reperfusion, a consequent protective effect that may also be important, even though the plasma concentration of apocynin decreases as a result of the metabolism. Parenchymal NOX and phagocyte NOX2 are identified as the main contributors to mild IRI in the kidney.

## 4. Materials and Methods

### 4.1. Animals

Male C57BL/6 (Charles River, Germany) and bone marrow chimera mice (see below) weighing 25.4 ± 3.8 g were maintained under standardized conditions (12:12 h light-dark cycle, 40–70% relative humidity, and constant ambient temperature of 22 ± 2 °C), with free access to standard rodent chow (Akronom Kft., Budapest, Hungary) and tap water. The experiments were approved by the Animal Ethics Committee of Semmelweis University and the Pest County Government Office (PE/EA/2202-5/2017). All procedures were performed in accordance with the Hungarian Law on the protection and welfare of animals and the EU Directive 2010/63/EU for animal experiments.

For testing the role of neutrophil granulocytes, bone marrow chimeras with a neutrophil-deficient Mcl1^flox/flox^LysM^Cre/Cre^ hematopoietic system were used [47,48]. Briefly, C57BL/6 recipients were irradiated with 11.5 Gy gamma rays using a ^137^Cs source and injected intravenously with unfractionated bone marrow cells from wild-type (WT) or Mcl1^flox/flox^LysM^Cre/Cre^ (referred to as Mcl-1^ΔMyelo^) mice on C57BL/6 background. The number of circulating neutrophils was counted by flow cytometry four weeks later. Neutrophils were defined as Ly6G-positive cells within a typical forward and side-scatter gate.

### 4.2. SiRNA Application

Lyophilized Stealth™ siRNAs were purchased from ThermoFisher Scientific. Stealth™ siRNA pools were used to target NOX4 (MSS224870, MSS224872, MSS203388), and XOR (MSS238716, MSS238717, MSS278864). Non-coding siRNA (siNC) was used as a negative control. Solutions for siRNA treatment were prepared by diluting 50 μg siRNA in 0.3 mL sterile physiological saline (Salsol A, Teva, Gödöllő, Hungary) before use. SiRNA was administered through the renal vein with retrograde hydrodynamic injection (HDI) 48 h before ischemia, as described earlier [49]. The retroperitoneum was opened above the renal vessels from a median laparotomy to place an occlusion clip (BH31, Aesculap, Center Valley, PA, USA) on the left renal pedicle. The aorta and the caval vein were clipped, and the renal vein was punctured with a 26-gauge needle to inject 0.3 mL of siRNA solution (NOX4 or XOR siRNA) into the kidney. The needle was kept in place for 5–10 s and then slowly removed, maintaining compression on the renal vein for 30 s with a piece of Gelaspon (Chauvin Ankerpharm, Rudolstadt, Germany). The aorta and vena cava clips were removed immediately after injection. Sham treatment was performed similarly with non-coding siRNA sequences.

### 4.3. Administration of Pharmacologic Inhibitors

Apocynin (A10809), a NOX inhibitor, and oxypurinol (O6881), an XOR inhibitor, were purchased from Sigma-Aldrich (Budapest, Hungary). Administration in vivo was carried out by pharmacokinetic properties, and plasma half-life of apocynin [50] and oxypurinol [51]. Apocynin was administered at 10 mg/kg body weight (BW) intravenously (i.v.) 15 min before ischemia and was repeated twice intraperitoneally (i.p.) right after and 120 min after ischemia. Oxypurinol was injected at 50 mg/kg BW i.v. 30 min before and at 50 mg/kg BW i.p. right after renal ischemia. Sham treatment consisted of equal volumes of saline.

### 4.4. Kidney Ischemia–Reperfusion

Unilateral renal IR with contralateral nephrectomy in one operation was carried out as described earlier [5]. During surgery, core body temperature was maintained using a heating pad (Supertech Ltd., Budapest, Hungary). 

Mice were anesthetized with ketamine (80 mg/kg BW, CP-Pharma Handelsgesellschaft mbH, Burgdorf, Germany) and xylazine (4 mg/kg BW, CP-Pharma Handelsgesellschaft mbH, Burgdorf, Germany) i.p. The left renal pedicle was clamped with two occlusion clips from a median laparotomy to ensure secure occlusion (BH31, Aesculap, Center Valley, PA, USA) for 15, 20 or 30 min. The right kidney was removed during ischemia. The same surgery was performed in sham-operated mice without clamping the renal pedicle. After surgery, Ceftriaxone (Rocephin, Roche Hungary Ltd., Budaörs, Hungary) was injected subcutaneously (s.c.) at 20 mg/kg to prevent infections.

### 4.5. Animal Sacrifice and Sample Collection

After renal ischemia, the animals were sacrificed at various times (1, 3, 6, 15, 24 or 48 h). Mice received heparin i.p. (5000 U/kg BW, Ratiopharm GmbH, Ulm, Germany) and were killed by cervical dislocation 3 min later. The chest was opened, and blood was collected from the thoracic cavity after cutting the vena cava superior. Plasma was immediately separated by centrifugation at 1500 g/10 min/4 °C. A total of 100 µL aliquots of the plasma were stored at −80 °C until use. Mice were perfused transcardially with cold (4 °C) physiological salt solution (Salsol A, Teva, Debrecen, Hungary) through the left ventricle. The kidney was cut into pieces for morphological and molecular investigations, snap-frozen in liquid nitrogen, and kept at −80 °C until use.

### 4.6. BUN Assay and Lcn-2 ELISA

Renal function was assessed by measuring plasma urea concentrations (Urea UV enzymatic, colorimetric kit, Diagnosticum Inc., Budapest, Hungary) according to the manufacturer’s protocol, and optical density was read at 340 nm (Victor3™ 1420 Multilabel Counter (PerkinElmer, Wallac Oy, Turku, Finland)). BUN values were calculated from the urea concentrations.

Plasma Lcn-2 protein concentration was assayed by the mouse Lipocalin-2/NGAL DuoSet ELISA Development kit (R&D Systems, Minneapolis, MI, USA). Briefly, 96-well plates (Nunc™ GmbH & Co. KG, Langenselbold, Germany) were coated with capture antibody, and non-specific binding sites were blocked with reagent diluent. Plasma samples were incubated in duplicates for 2 h, and the detection antibody was added. Next, Streptavidin-HRP was linked to the detection antibody, followed by a short incubation with TMB substrate (Sigma-Aldrich Chemie GmbH, Steinheim, Germany). Samples were washed between each step (5 times with 300 μL washing buffer) until the addition of the substrate solution. Optical density was read at 450 nm with wavelength correction set to 544 nm (Victor3™ 1420 Multilabel Counter (PerkinElmer, Wallac Oy, Turku, Finland)). Concentrations were calculated using a four-parameter logistic curve fit.

### 4.7. Renal Histology

Renal tissue samples fixed in 4% buffered formaldehyde (Molar Chemicals Ltd., Halásztelek, Hungary) were dehydrated and embedded in paraffin wax (FFPE) for histology and immunohistochemistry. Renal tissue injury was microscopically evaluated in periodic acid-Schiff (PAS)-stained kidney sections. Sections were scored in a blinded manner. The acute tubular injury was scored using a 0 to 4 scale, as described earlier [5]: 0 = normal morphology without lesion; 1 = tubular cell swelling, mild brush border damage, minimal or focal changes affecting less than 20% of the fields; 2 = moderately dilated tubules, moderate brush border loss, edematous tubular epithelial cells, focally weak nuclear staining, extension of the lesion to approx. 25% of the fields; 3 = vacuolization of tubular epithelial cells, loss of brush border, loss of nuclear staining, more severe tubular necrosis, extension of the lesion from 25% to 50% of the fields; 4 = total tubular necrosis, neutrophil granulocyte infiltration, no nuclear staining, dilated tubules, tubular cast formation in the lumen, extension of the lesion more than 50% of the fields. The ATN score was calculated as the mean of 10 fields of view. 

The renal neutrophil infiltration was evaluated by tissue microarray (TMA). For TMA, 2 mm diameter cylinders were cut in duplicate from each FFPE block with a computer-controlled punch press of the TMA Master Device (3DHISTECH Kft, Budapest, Hungary). Immunohistochemistry of myeloperoxidase (MPO) was executed on TMA sections of 70 samples. Four-µm thick sections mounted on adhesive glass slides were subjected to heat-induced epitope retrieval (HIER) in an electric pressure cooker (AVAIR IDA, YDB50-90D; Biofa Ltd., Veszprem, Hungary) using 12 min boiling at full pressure in a mixture of 0.1 m Tris-base and 0.01 m ethylenediamine tetraacetic acid (EDTA) (pH 9.0; Tris/EDTA). Then, overnight incubation with rabbit polyclonal anti-human myeloperoxidase (MPO) IgG (1:300, Agilent/Dako, Glostrup, Denmark) was followed by using the Novolink polymer kit, including the ‘post-primary block’ for 30 min, the peroxidase-labeled polymer for 60 min (RE7140-CE; Leica/NovoCastra, Newcastle upon Tyne, UK). Immunoreactions were revealed using a diaminobenzidine (DAB)-H_2_O_2_ chromogen substrate system for 5–8 min in brown color under microscopic control. Finally, the slides were counterstained with hematoxylin and coverslip-mounted after dehydration. Immunostained TMA slides were digitalized using a Pannoramic Scan instrument and were processed by Pannoramic Viewer v. 1.15 (3DHISTECH). Immunostaining was quantified by the CellQuant module of QuantCenter software v. 2.3 (3DHISTECH).

### 4.8. RNA Preparation

RNA was prepared as described previously [52]. RNA was extracted from the kidneys with TRI Reagent^®^ (Molecular Research Center, Inc., Cincinnati, OH, USA). RNA concentration and purity were checked with a NanoDrop 2000c spectrophotometer (Thermo Fisher Scientific, Waltham, MA, USA). All RNA samples had an absorbance ratio (260 nm/280 nm) above 1.8. To check RNA integrity, the samples were electrophoresed on 1% agarose gel (Invitrogen Ltd., Paisley, UK) in a Bio-Rad Wide mini-sub1cell GT system (Bio-Rad Laboratories Inc., Hercules, CA, USA). The RNA solutions were kept at −80 °C until use.

### 4.9. Quantitative Real-Time PCR Analysis for mRNA Expression in Renal Tissue

Lipocalin-2 (Lcn-2), xanthine oxidoreductase (XOR), NADPH oxygenase 2 (NOX2), NADPH oxygenase 4 (NOX4), the alpha subunit of cytochrome b (p22^phox^), nuclear factor erythroid 2–related factor 2 (NRF2), heme oxygenase 1 (HO-1), EGF-like module-containing mucin-like hormone receptor-like 1 (EMR1 or F4/80), tumor necrosis factor alpha (TNF-α) and glutathione peroxidase 3 (GPx3) mRNA expression were measured by double-stranded DNA (dsDNA) dye based real-time PCR [52]. Reverse transcription into cDNA was carried out by the High-Capacity cDNA Reverse Transcription Kit (Applied Biosystems, Foster City, CA, USA) according to the manufacturer’s protocol. In brief, 1 μg of total RNA was reverse-transcribed into cDNA with random hexamer primers at 37 °C for 2 h in a Bio-Rad iCycler™ Thermal Cycler (Bio-Rad Laboratories, Inc., Hercules, CA, USA). Expression levels were quantified by a Bio-Rad C1000™ Thermal Cycler with CFX96™ Optics Module real-time PCR system (Bio-Rad Laboratories, Inc., Singapore), using primer sequences from IDT (Table 1). Primers were designed by NCBI/Primer-BLAST online, except for the Gpx3 primer, adapted from Li et al. [41], and for the NRF2 primer, adapted from Hamada et al. [53]. All samples were measured in duplicates and mRNA expression was calculated using the relative quantification (ΔΔCq) method. The qPCR reactions were performed with Maxima™ SYBR^®^ Green qPCR Master Mix (Fermentas, St. Leon-Rot, Germany), and with SsoAdvanced™ Universal SYBR^®^ Green Supermix (Bio-Rad Laboratories, Inc., Hercules, CA, USA), according to the protocols.

### 4.10. Statistical Analysis

Results are expressed as mean ± standard error of the mean (SEM) unless otherwise indicated. One-way analysis of variance (ANOVA), followed by Dunnett’s multiple comparisons test, was used if Bartlett’s test verified the homogeneity of variances. Otherwise, Kruskal-Wallis ANOVA by ranks and Dunn’s test were used. Two-way ANOVA, followed by Sidak’s multiple comparisons test, was used when the effect of ischemia and neutrophil deficiency was evaluated. Two-way repeated measures ANOVA, followed by Sidak’s multiple comparisons test, was used for comparing the results of time–course experiments. The null hypothesis was rejected if *p* < 0.05. Statistical analysis was performed using GraphPad Prism 8 (GraphPad Software Inc., San Diego, CA, USA).

## 5. Conclusions

Neutrophils play a major role in injury following mild renal ischemia–reperfusion. Similarly, pharmacological inhibition of the local production of ROS by renal NOX enzymes provides protection after mild kidney damage, and a subsequent decrease in neutrophil infiltration may contribute to this protective effect. However, none of the above interventions can prevent moderate/severe ischemic injury. Xanthine oxidoreductase and the NOX4 isoform of NADPH oxidase alone do not substantially contribute to the pathogenesis of renal ischemia–reperfusion injury, regardless of severity. The main conclusion is that NADPH oxidases play a crucial role in mild renal ischemia–reperfusion injury, but in the case of a severe acute ischemia, the renal damage cannot be reduced by anti-NOX interventions.

## Figures and Tables

**Table 1:** Primers used for qPCR.

Target Gene	Forward Sequence (5′ to 3′)	Reverse Sequence (5′ to 3′)
18S	CTCAACACGGGAAACCTCAC	CGCTCCACCAACTAAGAACG
GAPDH	CCAGAATGAGGATCCCAGAA	ACCACCTGAAACATGCAACA
Lcn-2	AGGTGGTACGTTGTGGGC	CTGTACCTGAGGATACCTGTG
NOX2	TGCCACCAGTCTGAAACTCAA	AGCAAAGTGATTGGCCTGAGA
NOX4	CCAGAATGAGGATCCCAGAA	ACCACCTGAACCATGCAACA
p22^phox^	CGATCAGTGAGGACTTGCGA	CACACCTGCAGCGATAGAGT
XOR	GGCCATTTATGAAGCCTGTCA	GAAGTAGTGGAAGGGGTTCC
NRF2	CCTCACCTCTGCTGCAAGTA	GCTCATAGTCCTTCTGTCGCT
HO-1	ACAGAAGAGGCTAAGACCGC	GGCAGTATCTTGCACCAGG
TNF-α	AAATGGCCTCCCTCTCATCA	AGATAGCAAATCGGCTGACG
F4/80	TTTCCTCGCCTGCTTCTTC	CCCCGTCTCTGTATTCAACC
GPx3	CATCCTGCCTTCTGTCCCTG	CGATGGTGAGGGCTCCATAC

## Data Availability

The data presented in this study are available on request from the corresponding author.
